# Proteomic Identification and Quantification of Basal Endogenous Proteins in the Ileal Digesta of Growing Pigs

**DOI:** 10.3390/ani14132000

**Published:** 2024-07-07

**Authors:** Iris Elisa Ávila-Arres, Elba Rodríguez Hernández, Sergio Gómez Rosales, Tércia Cesária Reis de Souza, Gerardo Mariscal-Landín

**Affiliations:** 1 Posgrado en Ciencias de la Producción y de la Salud Animal, Facultad de Estudios Superiores Cuautitlán, Universidad Nacional Autónoma de México, Ciudad de México 04510, Mexico; iris_eaa@comunidad.unam.mx; 2 Centro Nacional de Investigación Disciplinaria en Fisiología y Mejoramiento Animal, INIFAP, Km 1 Carretera a Colón, Querétaro 76280, Mexico; rodriguez.elba@inifap.gob.mx (E.R.H.); gomez.sergio@inifap.gob.mx (S.G.R.); 3 Facultad de Ciencias Naturales, Universidad Autónoma de Querétaro, Av. de las Ciencias s/n, Querétaro 76230, Mexico; tercia@uaq.mx

**Keywords:** proteomics, basal endogenous losses, pigs, nitrogen-free diet, casein diet

## Abstract

**Simple Summary:**

As variations in the determination of basal endogenous losses can affect the estimation of the nutritional value of diets, this study used a proteomics approach to identify the composition of endogenous proteins in the ileal digesta of growing pigs fed with a nitrogen-free diet and a casein diet. The nitrogen-free diet increased the expression of proteins related to intestinal inflammation, the activation of innate antimicrobial host defense, cellular autophagy, and epithelial turnover in the ileal digesta. In contrast, the casein diet increased the proteins related to pancreatic and intestinal digestive secretions. These findings suggest that a casein diet could provide a more accurate estimation of basal endogenous losses.

**Abstract:**

The accurate estimation of basal endogenous losses (BEL) of amino acids at the ileum is indispensable to improve nutrient utilization efficiency. This study used a quantitative proteomic approach to identify variations in BEL in the ileal digesta of growing pigs fed a nitrogen-free diet (NFD) or a casein diet (CAS). Eight barrow pigs (39.8 ± 6.3 kg initial body weight (BW)) were randomly assigned to a 2 × 2 crossover design. A total of 348 proteins were identified and quantified in both treatments, of which 101 showed a significant differential abundance between the treatments (*p* < 0.05). Functional and pathway enrichment analyses revealed that the endogenous proteins were associated with intestinal metabolic function. Furthermore, differentially abundant proteins (DAPs) in the digesta of pigs fed the NFD enriched terms and pathways that suggest intestinal inflammation, the activation of innate antimicrobial host defense, an increase in cellular autophagy and epithelial turnover, and reduced synthesis of pancreatic and intestinal secretions. These findings suggest that casein diets may provide a more accurate estimation of BEL because they promote normal gastrointestinal secretions. Overall, proteomic and bioinformatic analyses provided valuable insights into the composition of endogenous proteins in the ileal digesta and their relationship with the functions, processes, and pathways modified by diet composition.

## 1. Introduction

Standardized ileal digestibility (SID) values of amino acids are commonly used to improve nutrient utilization efficiency [1,2]. Calculating SID values requires the determination of the amino acid basal endogenous losses (BEL) [3]. Regardless of the dietary composition or feeding conditions, the gastrointestinal tract constantly synthesizes and secretes proteins into the intestinal lumen for the digestion and absorption of dietary components, which also acts as the first line of immune defense in the intestine [4]. These endogenous secretions, including saliva, bile, gastric, pancreatic, and intestinal secretions, as well as mucoproteins, intestinal epithelial cell shedding, bacterial proteins, and plasma proteins [5,6], are quantitatively greater than those of dietary origin [7,8]. Approximately 60–80% of the proteins secreted into the intestinal lumen are digested and reabsorbed [8], whereas the non-reabsorbed amino acids from endogenous proteins are quantified at the end of the ileum of pigs to estimate the endogenous losses of amino acids [9]. In pigs, BELs of amino acids are typically determined by feeding them a nitrogen-free diet (NFD) or a diet containing highly digestible protein [10,11,12,13,14,15]. However, while these methodologies determine the BEL of amino acids and allow for the standardization of ileal digestibility values, discrepancies in method selection arise because of variations in the estimated endogenous amino acids, which can affect the accuracy of estimating the nutritional value of dietary ingredients [3,16].

Furthermore, these methodologies do not provide information on the origin of the endogenous nitrogen responsible for the variations in amino acids found in the ileal flow, making it challenging to explain inconsistencies between methodologies or within the same method. Although previous studies have identified and quantified the sources of endogenous secretions leading to the amino acids found in the ileal digesta using different methodologies [4,17,18], these methodologies can be costly or challenging to implement routinely [6,19].

With advances in biotechnology, proteomic approaches based on mass spectrometry (MS) have emerged. These approaches allow the identification and quantification of numerous proteins of endogenous origin and the assessment of the impact of different dietary factors on intestinal secretions, even without prior knowledge of the proteins present in the sample [20]. This study aimed to use proteomics to identify and quantify endogenous proteins secreted in the intestine and those remaining in the ileal digesta of pigs fed an NFD and a diet containing highly digestible proteins. Gene Ontology (GO) and Kyoto Encyclopedia of Genes and Genomes (KEGG) pathway enrichment analyses were performed on the identified proteins to elucidate their functions and metabolic pathways associated with the variations in basal endogenous losses observed with different diets, which will provide a better understanding of the response of intestinal physiology to diet.

## 2. Materials and Methods

### 2.1. Experimental Design and Collection of Digesta Samples

The Institutional Committee for the Care and Use of Experimental Animals of the Faculty of Veterinary Medicine and Zootechnics of the UNAM approved the animal experimental procedures (Protocol SICUAE.DC-2021/2-2). The committee adhered to the guidelines of the Official Mexican Standard (NOM-062-ZOO-1999) [21] and the International Guiding Principles for Biomedical Research Involving Animals [22].

Eight barrows (GP8 × Fertilis, PIC) with an initial body weight (BW) of 39.8 ± 6.3 kg were surgically implanted with a T cannula in the distal ileum [23]. The pigs were individually housed in metabolic cages with slat floors (0.80 m^2^) equipped with a feeder and a nipple drinker. The cages were kept in a room with a controlled temperature (22–24 °C) and natural light. The pigs were randomly allocated to the AB (*n* = 4) or BA (*n* = 4) sequence in a 2 × 2 crossover design. In the AB sequence, pigs received the nitrogen-free diet (NFD) in period 1 and the casein diet (CAS) in period 2, while in the BA sequence, pigs received CAS in period 1 and the NFD in period 2; there were a total of 8 observations per treatment. Each experimental period lasted seven days, with a seven-day washout interval between experimental periods to enable the animals to return to their normal metabolic state before starting the next period. Table 1 shows the composition and nutrient content of the experimental diets. Titanium dioxide was added to the diets as a digestibility marker. (Ileal digestibility data not present.) The experimental diets were balanced in nutrients, except protein, to meet the nutrient recommendations for growing pigs [24]. Feeding was restricted to 2.5 times the digestible energy requirement for maintenance of 110 kcal/kg BW^0.75^ [25]. Feed was provided twice daily, and the pigs had ad libitum access to water throughout the experiment. Digesta samples were collected for 12 h on days six and seven of each period using plastic bags tied to the cannula cylinder [26]. At the end of the collection, the samples from each pig and each period were homogenized, and a 50 mL aliquot was stored at −80 °C for proteomic analyses.

### 2.2. Proteomic Analysis of the Ileal Digesta

The ileal digesta samples were thawed (4 °C), homogenized, and centrifuged at 3000 rpm for 3 min. Supernatants were collected after centrifugation and the protein concentration of each sample was determined using a Bradford Protein Assay Quick Start kit (Bio-Rad Laboratories, Hercules, CA, USA) and a bovine serum albumin standard curve. Three technical replicates for each treatment were used for proteomic analyses to reduce individual variation. For the technical replicates, equiponderant proteins from the supernatant of each digesta sample were pooled per treatment and divided into three equal parts (*n* = 3) [27]. The supernatant proteins were precipitated with methanol–chloroform, and the resulting pellets was enzymatically digested using the iST Sample Preparation iST^®^ kit (PreOmics, Munich, Germany) according to the protocol established by the manufacturer. The resulting peptides were dried using a Savant DNA120 SpeedVac Concentrator (Thermo Fisher Scientific, Waltham, MS, USA) and then resuspended with “LC-load” reagent (PreOmics, Munich, Germany). An aliquot of 2.5 μL from a stock of alcohol dehydrogenase 1 (ADH1) from *Saccharomyces cerevisiae* (Uniprot, accession number P00330; 1 pmol/μL) was added as an internal standard to each sample to obtain a final concentration of 25 fmol/µL (final volume of 100 μL). Finally, samples were stored at −20 °C until LC-MS analysis.

Label-free identification and quantification by MS was carried out using the methodology described by Rios-Castro [28], with some modifications; briefly, tryptic peptides were separated on an HSS T3 C18 column (Waters, Milford, MA, USA; 75 μm × 150 mm, 100 Å pore size, 1.8 μm particle size) using a UPLC ACQUITY M-Class with mobile phase A (0.1% formic acid (FA) in H_2_O) and mobile phase B (0.1% FA in acetonitrile (ACN)) under the gradient 0 min 7% B, 121.49 min 40% B, 123.15 to 126.46 min 85% B, and 129 to 130 min 7% B, at a flow of 400 nL·min^−1^ and 45 °C. The spectrum data were acquired in a Synapt G2-S*i* mass spectrometer (Waters, Milford, MA, USA) using nano electrospray ionization and ion mobility separation (IM-MS) using a data-independent acquisition (DIA) approach through High-Definition Multiplexed MS/MS (HDMS^E^) mode. For the ionization source, parameters were set with the following values: 2.75 kV in the sampling capillary, 30 V in the sampling cone, 30 V in the source offset, 70 °C for the source temperature, 0.5 bar for the nanoflow gas, and 150 L·h^−1^ for the purge gas flow. Two chromatograms were acquired (low- and high-energy chromatograms) in positive mode in a range of *m*/*z* 50−2000 with a scan time of 500 ms. No collision energy was applied to obtain the low-energy chromatograms, while for the high-energy chromatograms, the precursor ions were fragmented in the transfer cell using a collision energy ramp from 19 to 55 eV.

The data generated in the mass spectrometer in *.raw format were analyzed and quantified using the Progenesis QI *v*3.0.3 proteomics software (Waters, Milford, CT, USA) against the *Sus scrofa* reference database downloaded from UNIPROT (https://www.uniprot.org/ accessed on 31 January 2023). Sequences were concatenated in reverse sense in the same *.Fasta file used to apply the target–decoy strategy to deliver false-positive estimations. The parameters for the protein identification were trypsin as the cutting enzyme and one missed cleavage site allowed; carbamidomethyl (C) as fixed modification; and oxidation (M), amidation (C-terminal), desamidation (Q, N), and phosphorylation (S, T, Y) as variable modifications. Fragment mass tolerance was set to 20 ppm and 10 ppm for peptides. The protein false discovery rate was set to <1%. All false-positive identifications (reversed proteins) and proteins with 1 peptide identified were discarded for subsequent analysis. Protein quantification was performed using the Top3 method. This method quantifies proteins by averaging the spectrometric signal of the three most intense tryptic peptides of each protein for quantification [29].

### 2.3. Statistical and Bioinformatic Analysis

Statistical analyses and data visualization were performed using normalized protein quantification values for each protein. A one-way ANOVA was used to perform the significance test to determine differences in protein abundance between treatments. Fold change (FC) was calculated as the CAS/NFD ratio. Proteins with a *p*-value < 0.05 and log_2_ FC greater than 0.5 or less than −0.5 were considered differentially abundant proteins (DAP). A heatmap and volcano plot were generated using the R v.4.2.2 software (http://www.R-project.org accessed on 2 June 2023). GO (http://geneontology.org/ accessed on 2 June 2023 ) and KEGG pathway (http://www.genome.jp/kegg/ accessed on 2 June 2023) enrichment analyses were performed to assess the functions and biological pathways associated with the proteins identified in the digesta using the online platform ShinyGO against the *Sus scrofa* database [30]. The GO terms and KEGG pathways were considered significantly enriched with a *p*-value < 0.05, corrected by FDR (FDR < 0.05). GO enrichment analysis was used to classify the proteins into biological processes (BPs), cellular components (CCs), and molecular functions (MFs).

## 3. Results

### 3.1. Proteomic Characterization of Endogenous Proteins in the Ileal Digesta

In total, 348 endogenous proteins were identified and quantified in the ileal digesta. Table 2 shows the 20 most abundant endogenous proteins found in the ileal digesta. These proteins accounted for approximately 40% of the endogenous proteins identified in the ileal digesta of growing pigs fed the NFD or CAS diets. A complete list of the identified proteins is provided in Supplementary Material Table S1.

**Figure 1:**
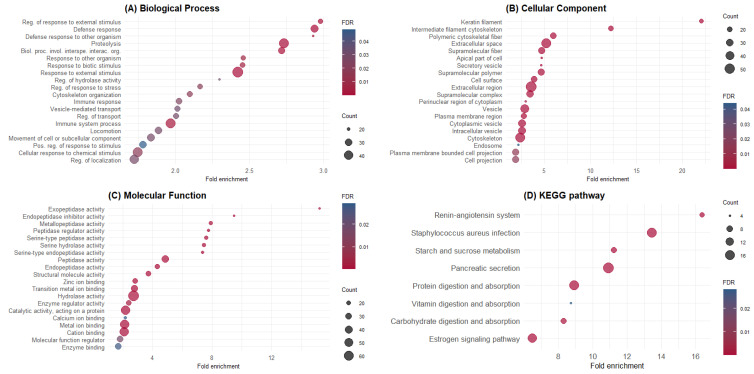
Gene Ontology and KEGG pathway enrichment analysis of ileal endogenous proteins. (**A**) Biological process; (**B**) cellular compartment; (**C**) molecular function; (**D**) KEGG pathways. The y-axis shows significantly enriched GO terms and pathways, whereas the x-axis denotes fold enrichment. The size of dots represents the number of genes within this term or pathway, and dots’ colors represent the enrichment FDR.

GO and KEGG enrichment analyses were performed using the 348 identified proteins. The top 20 GO terms enriched for BPs, CCs, and MFs are shown in Figure 1. Additionally, the proteins identified in pig digesta significantly enriched eight KEGG pathways (Figure 1D). GO terms and KEGG pathways were associated with the maintenance of gut epithelium and function. A list of the proteins in each GO term and KEGG pathway is available in the Supplementary Material Table S2.

### 3.2. Analysis of DAPs

**Figure 2:**
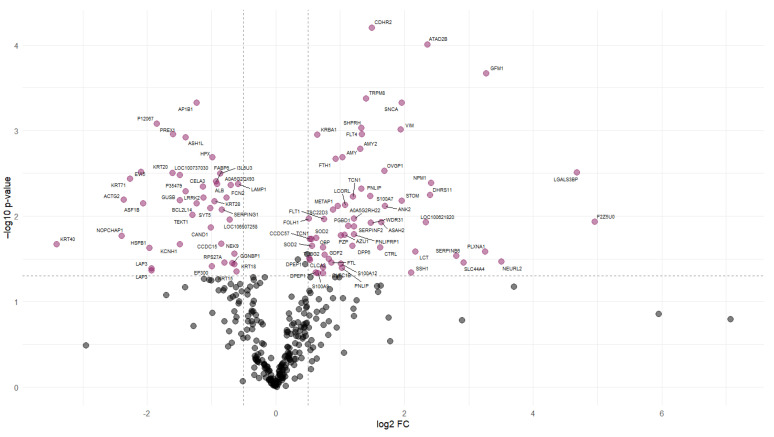
Volcano plot of differentially abundant proteins. Volcano plot representing each protein with a dot. The “x” axis represents the log_2_ FC between CAS and NFD and the “y” axis represents the *p*-value (−log_10_). The dashed lines indicate the significance limit in the *p*-value and FC. Gray dots represent proteins with non-significant changes in abundance. Colored dots represent proteins that significantly increase (**right**) or decrease (**left**) their abundance in the digesta of pigs fed CAS diets.

**Figure 3:**
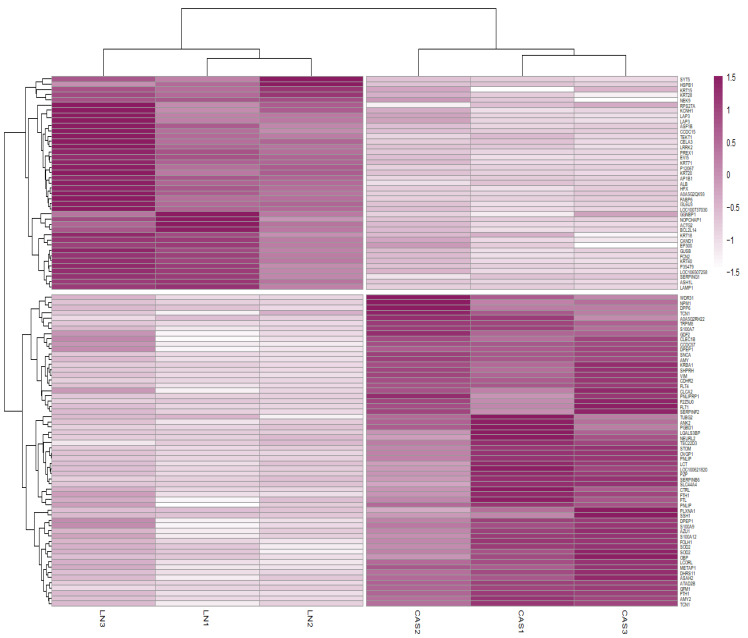
Heatmap of differentially abundant proteins (DAPs). Heatmap representing the hierarchical clustering of DAPs. Rows correspond to proteins and columns to samples. Colors represent the abundance values of proteins between NFD and CAS treatments.

Protein abundance analysis identified 101 DAPs between the NFD and CAS treatments. Figure 2 illustrates the changes in the abundance values of proteins and *p*-values using a volcano plot, and hierarchical clustering of the DAPs confirmed the presence of two distinct groups (Figure 3).

The analysis revealed 60 proteins with high abundance in the CAS treatment (Table 3) and 41 in the NFD treatment (Table 4).

**Figure 4:**
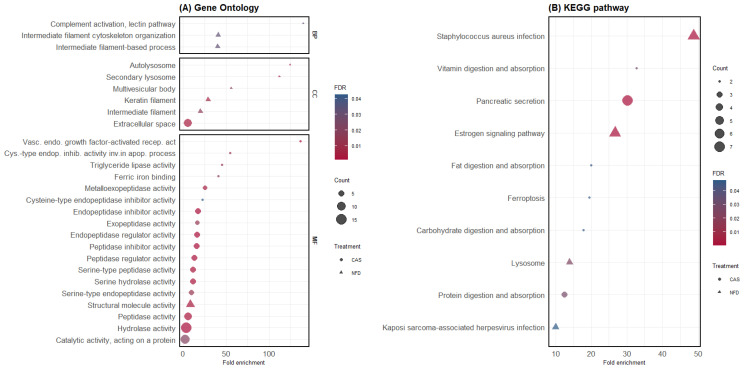
GO and KEGG pathway enrichment analysis of differentially abundant proteins. (**A**) GO enrichment analysis. The y-axis shows significantly enriched GO terms; BP: biological process, MF: molecular function, CC: cellular component (for each treatment (CAS and NFD)). The x-axis represents the fold enrichment, and the size of the dots represents the number of genes within each term. The colors of the dots represent the FDR enrichment. (**B**) KEGG pathway enrichment analysis. The x-axis displays the fold enrichment and the pathways enriched in CAS and NFD.

Separate GO and KEGG pathway enrichment analyses were performed for DAPs, which were highly abundant in the digesta of pigs fed CAS or NFD. Functional enrichment analysis of DAPs in the digesta of CAS-fed pigs revealed 17 GO terms significantly enriched for MFs and a CC term, cellular space (Figure 4A), and the KEGG pathway enrichment analysis revealed six significantly enriched pathways (Figure 4B). In contrast, DAPs in the NFD treatment revealed nine significantly enriched GO terms (Figure 4A) and four KEGG pathways (Figure 4B). The list of proteins within each GO term and KEGG pathway is available in the Supplementary Material Table S3.

## 4. Discussion

The quantification of basal endogenous losses and the factors that affect them is essential to improve nitrogen use efficiency [19]. A proteomic approach was utilized to identify and quantify the endogenous protein composition in the ileal digesta of pigs fed NFD and CAS. The identified proteins give rise to the amino acids determined by conventional methods in ileal digestibility studies. Therefore, the findings of this study represent an approach to identify the origin of endogenous losses modified by diet composition, complementing the available information about endogenous protein losses [31]. A large number of endogenous proteins were identified in the ileal digesta. Functional enrichment analysis is a method for assigning functional annotations to proteins and grouping them into GO terms and KEGG pathways, reducing the complexity of individual protein analysis. This analysis allowed for the identification of biological and metabolic changes in the intestine modified by dietary composition, providing deeper insights into the impacts of the diets on basal endogenous loss secretions.

The identified endogenous proteins were associated with metabolic and physiological functions of the intestine, including nutrient transport, digestion, absorption, and immune responses, similar to other proteomic studies in pigs [27,32,33,34,35]. The intestinal epithelium is exposed to various external stimuli, leading to the expression of proteins that interact with feed and microorganisms in the intestinal lumen [36]. In this study, we identified proteins associated with the response to external stimuli, including chemical, physical, and other organismal stimuli. In addition, processes related to the immune response, the regulation of localization, cellular transport, and proteolysis were also highlighted.

To maintain physiological functions and intestinal homeostasis, the intestine contains secretory cells responsible for the continuous release of significant quantities of proteins into the intestinal lumen [37]. The secretory cells in the intestinal epithelium include mucus-producing goblet cells, antimicrobial peptide-secreting Paneth cells, hormone-secreting enteroendocrine cells, and rare infection-mediating tuft cells. These epithelial cells provide the first line of chemical and physical defense against external factors to ensure a stable internal environment, supporting efficient digestion and absorption of nutrients [38]. These findings align with the results of the present study, which identified enriched terms associated with extracellular proteins and proteins secreted by vesicles. Notably, Annexin A4 (ANXA4), which promotes membrane fusion and is essential for exocytosis [39], was abundant in the digesta. Moreover, GO enriched terms were associated with catalytic, regulatory, or inhibitory enzymatic activity. At the molecular level, catalytic functions are indispensable for the regulation of inflammatory processes, extracellular tissue degradation, and intracellular particle breakdown in the intestine. Therefore, under physiological and pathological conditions, the gastrointestinal tract secretes a substantial quantity of proteases, including those originating from the pancreas and intestinal epithelium [40]. This study revealed a high abundance of proteases, such as Meprin A beta subunit (MEP1B), in the digesta. Meprins are metalloproteinases with a highly glycosylated domain that are abundantly expressed in the apical region of the ileal epithelial cells [41,42]. The Meprin A beta subunit regulates shedding of mucus secreted by goblet cells through proteolytic cleavage of MUC2, which leads to its release [42,43]. Although an increase in mucus secretion has been observed in animals fed an NFD [44], no differences were detected in the proteins associated with mucus secretion that were identified in this study (MUC2, MUC13, MEP1B, KLK1, ADAM10, CLCA1, and FCGBP) [43].

Furthermore, the intestinal secretion of endogenous protease inhibitors helps maintain intestinal homeostasis and protects biologically important proteins, such as immune-active proteins [40]. Alpha-2 macroglobulin (A2M), identified in the digesta, inhibits endopeptidases of all catalytic types, preventing the degradation of endogenous biologically active proteins without interfering with the active site of the protease [45]. In the intestine, immunoglobulin production is essential for protecting the epithelial barrier. Through proteomic analyses, immunoglobulins and their fragments have been identified as some of the most abundant endogenous proteins in the digesta [32,33,34], consistent with our findings, where different immunoglobulin fragments were identified. Additionally, Ig lambda chain C and IgA constant regions were the two most abundant proteins in the digesta. Despite the observed reduction in immunoglobulin production under nutrient deprivation [46], no differences were observed between the treatments in our study, likely due to the increased starch availability in NFD, which could have modulated the microbiota and increased immunoglobulin production [46].

Enzymatic secretion is one of the main components of endogenous loss in the ileum [5,19]. In this study, the proteins identified in the digesta enriched pathways associated with the digestion and absorption of proteins, carbohydrates, lipids, vitamins, and minerals, similar to previous proteomics findings in the digesta of growing pigs [35]. Of these, 19 corresponded to pancreatic secretions. Research in pigs has shown that pancreatic secretions typically account for approximately 5% of endogenous nitrogen secreted in the intestine [5]. Diet composition alters enzyme secretion, and in starch-rich diets, an increase in salivary and pancreatic amylases as well as sucrase-isomaltase and maltase-glucoamylase in the intestine has been observed [47]. In this study, maltase-glucoamylase and sucrase-isomaltase were the most abundant proteins in the digesta, although no differences were observed between the treatments. The absence of differences could be due to a compensatory increase in maltase-glucoamylase in pigs fed the NFD, as in situations of decreased amylase activity, its secretion increases to hydrolyze starch [33]. This is consistent with the reduction in pancreatic amylase (AMY and AMY2) levels observed in NFD-fed pigs. Furthermore, nutrient deprivation has been associated with a decrease in the synthesis and secretion of pancreatic enzymes [46,47,48], similar to the findings of this study, where NFD-fed pigs showed decreased secretion of pancreatic proteases and lipases, along with other intestinal peptidases and proteins involved in the digestion and absorption of vitamins and minerals.

High dietary carbohydrate levels have been associated with an increased abundance of pathogenic bacteria [49], potentially promoting the enrichment of the *S. aureus* infection pathway in the digesta and increasing this pathway in pigs fed NFD. Within this pathway, the protein Ficolin-2 (FCN2), which is overexpressed in the digesta of NFD-fed pigs, participates in the complement activation lectin pathway by binding to lipoteichoic acid present in the cell walls of Gram-positive bacteria, including *S. aureus* [50,51]. The complement activation lectin pathway is crucial for innate antimicrobial host defense [52]. In addition to pathogens, lectin receptors recognize endosome derivatives and damage-associated molecular patterns in extracellular tissues [49,51]. Therefore, along with the enrichment of the autolysosome and lysosome pathways, these results suggest an increase in cellular autophagy in pigs fed NFD. Nutrient deficiency, including amino acid deficiency, decreases mTOR activity. mTOR phosphorylates the autophagy-initiating complex and inhibits autophagosome biogenesis.

Consequently, during prolonged periods of starvation, cellular autophagy increases, and autolysosome degradation products are recycled to maintain homeostasis and regulate cellular functions [53]. Furthermore, autophagy is promoted by the overgrowth of pathogenic bacteria [53]. These factors compromise the function of the intestinal barrier by stimulating a pro-inflammatory environment that increases cell renewal and preserves tissue integrity [50,51].

Intestinal epithelial cells exhibit a rapid turnover rate characterized by migration, differentiation, and cell renewal every 3–5 days, which is crucial for protecting integrity and maintaining intestinal functions [54,55,56]. Previous studies have identified epithelial cell desquamation as a major source of endogenous proteins in the ileum [5]. In this study, the proteins identified in the digesta enriched terms associated with the cytoskeleton, with keratin being the most abundant. Keratin and keratin filaments are resistant to cleavage by proteolytic enzymes because they are stabilized by numerous cross-linked disulfide bonds [57]. In the intestinal epithelium, undifferentiated crypt cells express K18, whereas villus cells express K20 [58,59]. The abundance of these keratins in the digesta of pigs fed the NFD suggests an increase in intestinal epithelial cell turnover [60], which is consistent with previous findings demonstrating increased epithelial desquamation in animals fed NFD.

The increased rate of intestinal renewal may explain the enrichment of the estrogen signaling pathway in the digesta. This pathway is crucial for regulating epithelial cell proliferation and differentiation in the intestine, where estrogen acts as a transcription regulator [61,62]. Additionally, this pathway regulates the electrolyte balance and contributes to intestinal HCO_3_ secretion [63]. In NFD-fed pigs, this pathway was upregulated compared to CAS-fed pigs. Moreover, the high abundance of leucine-rich protein (LRRIQ3), which is implicated in intestinal repair [50], in the digesta of NFD-fed pigs suggests an increase in cell renewal. The presence of EP300, PREX1, and RPS27A proteins, which enrich the Kaposi’s sarcoma-associated herpesvirus infection pathway and growth differentiation factor 2 (GDF2), which stimulates cell differentiation, proliferation, and migration in enterocytes, intestinal stem cells, and goblet cells under conditions of inflammation [64,65,66], supports the hypothesis of increased intestinal epithelial cell shedding in NFD-fed pigs.

In the gastrointestinal tract, the renin–angiotensin system regulates the intestinal environment by modulating various physiological processes such as gastrointestinal motility, fluid secretion, and absorption, as well as the uptake of peptides, amino acids, glucose, and sodium [67,68]. Angiotensin-converting enzyme (ACE2), a cell membrane-bound carboxypeptidase predominantly expressed in the ileum, is essential for the function of the sodium-dependent amino acid transporter B(0)AT1 [69]. Previous proteomic studies conducted on the intestines of pigs have observed the enrichment of this pathway in the digesta [35], a finding consistent with the results of the present study. Additionally, it has been noted that endogenous secretions exhibit increased ACE inhibitory bioactivity [70], suggesting a potential mechanism of intestinal homeostasis aimed at regulating intestinal secretions.

## 5. Conclusions

In conclusion, the proteomic analyses performed in this study enabled the identification of endogenous proteins in the ileal digesta. The findings suggest that pigs fed a casein diet could be a better alternative for basal endogenous loss estimation due to promoting normal intestinal secretion and maintaining intestinal health. Basal endogenous losses can impact the nutrient requirements of protein and amino acids due to the metabolic cost associated with the synthesis and turnover of endogenous proteins in the intestine. Therefore, identifying endogenous proteins could significantly enhance the development of nutritional strategies aimed at maximizing nutrient utilization in pigs. While label-free proteomics sacrifices some precision, this approach could be considered a complementary and cost-effective method for characterizing and quantifying endogenous proteins in the ileal digesta under different feeding conditions. Comprehension of the factors that influence endogenous ileal losses is essential for improving nutrient utilization efficiency.

## Figures and Tables

**Table 1:** Composition and nutrient content of experimental diets.

	NFD ^1^	CAS
Ingredients, g/kg		
Cornstarch	795.4	615.4
Casein		180
Dextrose	100	100
Soybean oil	30	30
Cellulose	40	40
Calcium carbonate	5	5
Orthophosphate	19	19
Salt	4	4
Potassium bicarbonate	0.5	0.5
Magnesium oxide	0.1	0.1
Titanium dioxide	4	4
Vitamin mineral premix ^2^	2	2
Calculated nutrient content		
Metabolizable energy, Kcal, Kg	3481	3473
Crude protein, %	0	16
Crude fiber, %	2.86	2.83

^1^ NFD = nitrogen-free diet, CAS = casein diet; ^2^ provided per kilogram of diet: Co 0.42 mg, Cu 8.4 mg, Fe 70 mg, I 0.56 mg, Mn 21 mg, Se 0.18 mg, Zn 84 mg, vitamin A 10, 1000 IU, vitamin D3 2000 IU, vitamin E 120 mg, vitamin K 8 mg, thiamine 0.35 mg, riboflavin 10 mg, pyridoxine 6 mg, cyanocobalamin 0.05 mg, niacin 60 mg, pantothenic acid 35 mg, folic acid 1.5 mg, and biotin 0.3 mg.

**Table 2:** Top 20 most abundant endogenous proteins in the digesta.

Accession	GEN	Protein Name	TOP NFD	TOP CAS
P01846		Ig lambda chain C region	1	1
P00761		Trypsin	2	2
A0A287A042	MGAM	Maltase-glucoamylase	5	4
P09955	CPB1	Carboxypeptidase B	6	3
P08419	CELA2A	Chymotrypsin-like elastase family member 2A	3	10
A0A5G2QEP8	LRRIQ3	Leucine-rich repeats and IQ motif containing 3	4	11
K7GMF9	ANPEP	Aminopeptidase	7	6
A0A5G2RGB6	MEP1B	Meprin A subunit B	9	5
C6L245	try	Putative trypsinogen	11	7
A0A287AYJ2	ANXA4	Annexin A4	10	14
A0A287B626		IgA constant region	12	13
F1RRW5	ACE	Angiotensin-converting enzyme	16	8
P56729	SI	Sucrase-isomaltase intestinal	14	16
A0A287BPD6	MME	Neprilysin	13	20
F1SUW2	CTRC	Chymotrypsin-C	15	
A0A5G2QAG3	LAP3	Cytosol aminopeptidase	8	
P09954	CPA1	Carboxypeptidase A1	18	
A0A287AT48	LOC100153899	Alpha-1-antichymotrypsin 2		12
A0A5G2RDG3	NAALADL1	N-acetylated alpha-linked acidic dipeptidase like 1	17	
F1STN0	SMPDL3B	Sphingomyelin phosphodiesterase acid-like 3B		18
P80310	S100A12	Protein S100-A12		15
F1SCC9	LOC106504545	SERPIN domain-containing protein	20	
I3LHI7	LOC100621820	Peptidase S1 domain-containing protein		9
K7GMV8	ENPP3	Ectonucleotide pyrophosphatase/phosphodiesterase 3	19	
I3LCF8	CTRL	Chymotrypsin like		17
A0A286ZTL5	WDR31	WD repeat domain 31		19

**Table 3:** Proteins with high abundance in the digesta of pigs fed CAS diets.

Accession	GEN	Protein Name	log_2_ FC
F2Z5U0		Ubiquitin-40S ribosomal protein S27a	4.96
A0A287A604	LGALS3BP	Galectin-3-binding protein	4.68
F1SC72	NEURL2	Neuralized E3 ubiquitin protein ligase 2	3.51
A0A287B4A9	GFM1	G elongation factor mitochondrial 1	3.27
A0A287A6D8	PLXNA1	Plexin A1	3.26
A0A2C9F351	SLC44A4	Choline transporter-like protein	2.92
A0A286ZRF3	SERPINB6	Serpin family B member 6	2.80
I3LUP6	NPM1	Nucleophosmin	2.42
F1S1B9	DHRS11	Dehydrogenase/reductase 11	2.40
A0A287BGV4	ATAD2B	ATPase family AAA domain containing 2B	2.36
I3LHI7	LOC100621820	Peptidase S1 domain-containing protein	2.34
A0A5G2QS59	LCT	LPH hydrolase	2.18
F1RGB0	SSH1	Protein-serine/threonine phosphatase	2.11
A0A5G2R2I6	SNCA	Alpha-synuclein	1.96
A0A287B310	STOM	Stomatin	1.96
A0A5S6H025	VIM	Vimentin	1.95
A0A287AUI5	ANK2	Ankyrin-2	1.70
A0A286ZZ91	OVGP1	Oviduct-specific glycoprotein	1.69
A0A5G2Q9X4	ASAH2	Neutral ceramidase	1.64
I3LCF8	CTRL	Chymotrypsin like	1.63
A0A286ZUV2	CDHR2	Cadherin-related family member 2	1.50
A0A286ZTL5	WDR31	WD repeat domain 31	1.48
F1SFU5	S100A7	EF-hand domain-containing protein	1.47
F1SM15	TRPM8	Transient receptor potential cation channel subfamily M member 8	1.40
F1S5Q6	FLT4	Vascular endothelial growth factor receptor 3	1.34
A0A286ZWA9	SHPRH	SNF2 histone linker PHD RING helicase	1.33
P00591	PNLIP	Pancreatic triacylglycerol lipase	1.33
P00690	AMY2	Pancreatic alpha-amylase	1.31
A0A5G2RH22		L1 transposable element	1.22
A0A287B9B3	SERPINF2	Alpha-2-antiplasmin isoform X2	1.22
F6Q1W0	PNLIPRP1	Triacylglycerol lipase	1.22
P17630	TCN1	Transcobalamin-1	1.20
A0A5G2R7I6	DPP6	Dipeptidyl peptidase like 6	1.20
F1S1R8	PGBD1	PiggyBac transposable element derived 1	1.13
A0A287BIU6	LCORL	Ligand-dependent nuclear receptor co-repressor like	1.08
P80015	AZU1	Azurocidin	1.07
I3LSA5	AMY	Alpha-amylase	1.04
A0A5G2RJR8	PNLIP	Triacylglycerol lipase	1.03
P80310	S100A12	Protein S100-A12	1.02
A0A287A1B4	PZP	Pregnancy zone protein-like	1.02
A0A287BQW1	METAP1	Methionine aminopeptidase	0.97
P19130	FTH1	Ferritin heavy chain	0.93
A0A287AP88	TSC22D3	TSC22 domain family protein 3	0.89
P19133	FTL	Ferritin light chain (Fragment)	0.87
F1SEL6	GDF2	Growth Differentiation Factor 2	0.83
A0A287BHZ2	TUBG2	Tubulin gamma chain	0.76
F1RSU5	FLT1	Receptor protein-tyrosine kinase	0.75
A0A287AL10	FTH1	Ferritin	0.74
P81245	OBP	Odorant-binding protein	0.74
I3LNV9	CLEC1B	C-type lectin domain family 1 member B	0.73
K7GME6	S100A9	S100 binding calcium-binding protein A9	0.66
A0A287AES3	KRBA1	KRAB-A domain containing 1	0.65
P28768	SOD2	Superoxide dismutase [Mn]_ mitochondrial	0.63
I3L719	DPEP1	Dipeptidase	0.62
A0A287A4Z2	SOD2	Superoxide dismutase	0.57
A0A287A0W9	TCN1	Transcobalamin-1	0.56
A0A287AIG5	CCDC57	Coiled-coil domain containing 57	0.53
F1S4C6	CLCA2	Chloride channel accessory 2	0.53
P22412	DPEP1	Dipeptidase 1	0.52
O77564	FOLH1	Glutamate carboxypeptidase 2	0.52

**Table 4:** Proteins with high abundance in the digesta of pigs fed NFD.

Accession	GEN	Protein Name	log_2_ FC
F1RN44	LAMP1	associated lysosomal-associated membrane protein 1	−0.59
A0A286ZYN0	KRT15	Keratin 15	−0.61
A0A5G2RBD3	KRT18	Keratin 18	−0.64
A0A5G2QV66	NEK9	NIMA-related kinase 9	−0.64
F1RZR1	GGNBP1	Ubiquitin_3 domain-containing protein	−0.67
A0A5G2QX93		DUF1725 domain-containing protein	−0.70
A0A287A4P2	LOC106507258	IF rod domain-containing protein	−0.72
A0A287BQR3	FCN2	Ficolin-2	−0.77
A0A287AZA7	RPS27A	Ubiquitin-40S ribosomal protein S27a	−0.80
A0A5K1UE53	SERPING1	Serpin family G member 1	−0.84
F1S793	CCDC15	Coiled-coil domain containing 15	−0.84
I3L6U3		Uncharacterized protein	−0.86
P08835	ALB	Albumin	−0.91
P10289	FABP6	Gastrotropin	−0.92
F1RXG2	KRT28	Keratin 28	−0.95
F1RMN7	HPX	Hemopexin	−0.98
I3L9U8	EP300	Histone acetyltransferase	−0.99
A0A5G2RAC7	CAND1	Cullin associated and neddylation dissociated 1	−1.01
A0A5K1V9N9	SYT5	Synaptotagmin 5	−1.02
F1SHN7	LRRK2	Non-specific serine/threonine protein kinase	−1.12
A0A287BD64	CELA3	Peptidase S1 domain-containing protein	−1.13
F1SQ67	BCL2L14	BCL2 like 14	−1.23
A0A286ZWT0	AP1B1	AP complex subunit beta	−1.23
F1RGN9	TEKT1	Tektin	−1.29
A0A5G2RMY5	ASH1L	ASH1-like histone lysine methyltransferase	−1.40
P35479		Leukocyte cysteine proteinase inhibitor 1	−1.40
A0A287ACY3	KCNH1	Potassium voltage-gated channel subfamily H member 1	−1.49
A0A287AHM5	LOC100737030	IF rod domain-containing protein	−1.49
A0A2C9F3C0	GUSB	Beta-glucuronidase	−1.49
A0A286ZUE0	PREX1	Phosphatidylinositol-3_4_5-trisphosphate dependent Rac exchange factor 1	−1.60
A0A5G2QPZ4	KRT20	Keratin_ type I cytoskeletal 20	−1.60
P12067		Lysozyme C-1	−1.85
A0A5G2QAG3	LAP3	Cytosol aminopeptidase	−1.93
P28839	LAP3	Cytosol aminopeptidase	−1.93
A0A2C9F366	HSPB1	Heat shock protein beta-1	−1.96
B6DX84	ASF1B	ASF1B	−2.06
A0A286ZM40	EVI5	Ecotropic viral integration site 5	−2.09
A0A287A2G9	KRT71	Keratin 71	−2.26
A0A286ZWJ1	ACTG2	Actin gamma 2_ smooth muscle	−2.36
A0A287B664	NOPCHAP1	NOP protein chaperone 1	−2.40
A0A287ARK0	KRT40	Keratin 40	−3.41

## Data Availability

The mass spectrometry proteomics data have been deposited in the ProteomeXchange Consortium via the PRIDE (https://www.ebi.ac.uk/pride/ accessed on 24 May 2024) partner repository with the dataset identifier PXD052560.

## Supplementary Materials

The following supporting information can be downloaded at https://www.mdpi.com/article/10.3390/ani14132000/s1: Table S1. Abundance of endogenous proteins identified in the digesta of growing pigs fed NFD and CAS diets; Table S2. Gene Ontology and KEGG pathway enrichment analysis of ileal endogenous proteins. Biological process (BP), cellular compartment (CC), molecular function (MF), and KEGG pathways; Table S3. Gene Ontology (GO) and KEGG pathway enrichment analysis of differentially abundant proteins (DAPs). Biological process (BP), molecular function (MF), and cellular component (CC).
